# Additional evidence for the 'wimp SNP' concept of carcinogenesis

**DOI:** 10.17179/excli2017-947

**Published:** 2017-11-20

**Authors:** Hermann M. Bolt

**Affiliations:** 1 IfADo, Leibniz Research Centre for Working Environment and Human Factors, Dortmund

## ⁯

Dear Editor,

In 2011 Klaus Golka and colleagues from Dortmund University published the wimp SNP concept of carcinogenesis (Golka et al., 2011[8]). According to this concept, individual genetic variants, usually SNPs, confer only a small cancer risk. However, specific combinations of high risk variants may interact, leading to a much higher risk of affected individuals. Although Golka and colleagues established this concept originally for urinary bladder cancer, it may in principle apply for all types of cancer and any polygenic disease.

Until recently the wimp SNP concept was not much more than a fascinating hypothesis without comprehensive experimental proof. However, with the publication of a recent study including totally more than 10,000 cases and controls the situation has changed (Selinski et al., 2017[23]). Selinski and colleagues identified a statistical interaction of four high risk variants. Each of the individual high risk variants leads to an odds ratio of only 1.1-1.3. However, individuals that carry all four variants have a 2.6-fold increased risk. The four sequences, whose high risk variants interact, are a sequence near APOBEC3A, an exon of SLC14A1, an intron of UGT1A and a variant near CCNE1. Unfortunately, too little is known about each individual variant to understand the mechanism why they interact. However, the fact that wimp SNPs interact to cause high odds ratios has been confirmed. 

This progress has been made possible by numerous studies, mostly genome-wide association studies that have identified the individual high risk variants (e.g. Selinski, 2012[20], 2014[21][22]; Rafnar et al., 2009[15], 2011[17], 2014[16]; Kiemeney et al., 2008[12], 2010[11]; Garcia-Closas et al., 2011[6]; Figueroa et al., 2014[4], 2016[3]; Rothman et al., 2010[18]; Schwender et al., 2012[19]; Golka et al., 2011[8]). Currently, most studies on genetic polymorphisms in human disease still focus on individual variants (Huang et al., 2016[10]; Pellé et al., 2016[14]; Anvar et al., 2011[1]; Hashemi et al., 2015[9]; Liaqat et al., 2015[13]; Chu et al., 2016[2]; Fujihara et al., 2016[5]; Geller et al., 2016[7]). However, the recent study of Selinski et al. (2017[23]) has shown that identification of the most powerful interactions of individual SNPs is an attractive perspective of the post-GWAS era.
